# Drug Design, Synthesis and Biological Evaluation of Heterocyclic Molecules as Anti-Inflammatory Agents

**DOI:** 10.3390/molecules27041262

**Published:** 2022-02-14

**Authors:** Jignasa Savjani, Bhavesh Variya, Snehal Patel, Suja Mulamkattil, Harsh Amin, Shital Butani, Ahmed Allam, Jamaan Ajarem, Harsh Shah

**Affiliations:** 1 Department of Pharmaceutical Chemistry, Institute of Pharmacy, Nirma University, S.G.Highway, Ahmedabad 382481, India; bcvariya@gmail.com (B.V.); snehal.patel@nirmauni.ac.in (S.P.); 13mph405@nirmauni.ac.in (S.M.); harshamin143@gmail.com (H.A.); shital.butani@nirmauni.ac.in (S.B.); 2 Faculty of Pharmacy and Pharmaceutical Sciences, University of Alberta, Edmonton, AB T6G 2R3, Canada; 3 Zoology Department, Faculty of Science, Beni-Suef University, Beni-Suef 65211, Egypt; ahmed.aliahmed@science.bsu.edu.eg; 4 Zoology Department, College of Science, King Saud University, Riyadh 11451, Saudi Arabia; jajarem@ksu.edu.sa; 5 J-Star Research Inc., 6 Cedar Brook Drive, Cranbury, NJ 08512, USA; harsh.shah@jstar-research.com

**Keywords:** anti-inflammatory agents, indomethacin, COX-2 inhibitors, gastrointestinal safety study, molecular modelling

## Abstract

Non-steroidal anti-inflammatory drugs (NSAIDs) are generally utilized for numerous inflammatory ailments. The long-term utilization of NSAIDs prompts adverse reactions such as gastrointestinal ulceration, renal dysfunction and hepatotoxicity; however, selective COX-2 inhibitors prevent these adverse events. Various scientific approaches have been employed to identify safer COX-2 inhibitors, as in any case, a large portion of particular COX-2 inhibitors have been retracted from the market because of severe cardiovascular events. This study aimed to develop and synthesize a novel series of indomethacin analogues with potential anti-inflammatory properties and fewer side effects, wherein carboxylic acid moiety was substituted using DCC/DMAP coupling. This study incorporates the docking of various indomethacin analogues to detect the binding interactions with COX-2 protein (PDB ID: 3NT1). MD simulation was performed to measure the stability and flexibility of ligand–protein interactions at the atomic level, for which the top-scoring ligand–protein complex was selected. These compounds were evaluated in vitro for COX enzymes inhibition. Likewise, selected compounds were screened in vivo for anti-inflammatory potential using the carrageenan-induced rat paw oedema method and their ulcerogenic potential. The acute toxicity of compounds was also predicted using in silico tools. Most of the compounds exhibited the potent inhibition of both COX enzymes; however, **3e** and **3c** showed the most potent COX-2 inhibition having IC50 0.34 µM and 1.39 µM, respectively. These compounds also demonstrated potent anti-inflammatory potential without ulcerogenic liability. The biological evaluation revealed that the compound substituted with 4-nitrophenyl was most active.

## 1. Introduction

Non-selective NSAIDs and selective COX-2 inhibitors are a group of medicines used to relieve pain fever and reduce inflammation. Inflammation is an immediate consequence of tissue damage because of which the entire procedure of metabolic activities moves towards the catabolic side. There is an increase in the synthesis of prostaglandins as a result of inflammation [1]. Many heterocyclic molecules have been discovered for inflammation, but they all work in the same way, by blocking cyclooxygenase enzymes. These enzymes are responsible for the synthesis of prostaglandins, which are involved in different processes such as inflammation, blood flow and the formation of blood clots. The COX enzyme has two isoforms, namely, COX-1 and COX-2. Gastrointestinal adverse effects observed with non-selective NSAIDs are due to the inhibition of COX-1 enzyme. With the inhibition of COX-1, there is a decrease in the synthesis of prostaglandins responsible for cytoprotection2 [2,3]. Therefore, the long-term use of NSAIDs leads to severe gastric ulceration, hepatotoxicity and renal dysfunction. NSAIDs’ therapeutic benefits are due to the inhibition of the COX-2 enzyme, while the adverse effects are observed due to COX-1 inhibition. There are reports which confirm the potential benefits of COX-2 inhibitors over nonselective NSAIDs. Selective cyclooxygenase II inhibitors were discovered to reduce the side effects associated with NSAIDs. However, most selective COX-2 inhibitors were retracted from the market due to severe cardiovascular adverse effects [4,5].

The undesirable cardiovascular side effects identified with particular cyclooxygenase 2 inhibitors include an elevated TXA2 level [6]. Kalgutkar et al. developed different analogues of traditional non-steroidal anti-inflammatory drugs. This research group prepared different meclofenamic acid and indomethacin analogues, which demonstrated better activity towards COX-2 inhibition. Hence, as per the literature and SAR studies of the indomethacin analogues, we designed and synthesized different amide linkers [7]. Previously, we synthesized different derivatives of mefenamic acid and identified their COX inhibition potential and anti-inflammatory efficacy using in vitro enzyme assays and in vivo animal models, respectively. These novel mefenamic acid derivatives revealed better anti-inflammatory potential than the classical NSAIDs [8]. A few reports uncovered that derivatives designed from the classical NSAIDs showed better outcomes in terms of anti-inflammatory efficacy and non-ulcerogenic potential as well [7,9,10]. Looking into the advantages of selective COX-2 inhibitors encouraged us to design novel heterocyclic molecules with more potent anti-inflammatory activity. Consequently, we employed the design and development of amide derivatives of indomethacin as potential anti-inflammatory agents.

## 2. Materials and Methods

### 2.1. Molecular Docking Studies

Molecular docking was performed to investigate the ligand’s binding interaction with the COX-2 enzyme. SYBYL-X 1.2 and GOLD 5.2 software were used for the docking study. Energy minimization was performed using a conjugate gradient algorithm with a gradient convergence value of 0.01 kcal/mol Å. The Gasteiger–Hückel method was utilized for calculating partial atomic charges. The ligands were docked with the COX-2 enzyme obtained from PDB ID- 3NT1, having a resolution of 1.73 Å [11].

### 2.2. MD Simulation

MD simulations were utilized to evaluate the stability and flexibility of ligand–protein interactions at the atomic level using the highest scoring ligand–protein complexes in molecular docking calculations. GROMACS (version 2020.1) was used to carry out the MD simulations. The proteins’ topology was generated using the CHARMM36 force field (February 2021) and the TIP3P water model. The CGenFF server was used to retrieve ligand topologies. After neutralization, MD simulations were run in a dodecahedron box. The steepest descent minimization algorithm was used to perform energy minimizations. NVT and NPT equilibrations took one ns, followed by 10 ns of MD simulations at 1 bar and 300 K reference pressure and temperature.

### 2.3. Acute Toxicity Prediction Using PASS Online Software

The GUSAR (General Unrestricted Structure–Activity Relationships) online module was used for the in silico acute toxicity prediction. GUSAR software predicts the probable toxicity based on a database of around 10,000 chemical structures in the software library. QSAR (Quantitative Structure–Activity Relationship) analysis was employed for the prediction of the LD_50_ (log10 (mmol/kg) of a novel chemical entity. The analysis considers the different routes of administration viz. oral, subcutaneous, intravenous and intraperitoneal routes [12,13].

### 2.4. Synthesis of Indomethacin Derivatives (***3a**–**3i***)

Synthesis of indomethacin derivatives was performed in the borosilicate glassware. REMI rota mantle and magnetic stirrers were utilized to reflux and mix the reaction mixtures. Residual solvent was recovered using a rotary vacuum evaporator (Buchi type). The melting point was estimated employing a paraffin oil bath or digital melting point apparatus (VEEGO partnership). Precoated silica gel TLC plates (MERCK) were used to monitor the reaction’s progress, and a UV chamber was utilized to detect product formation. IR spectra were recorded on JASCO FTIR by the KBr scattering process. 1H NMR and 13C NMR spectra were recorded on a BRUKER 400 MHz instrument utilizing TMS as an internal standard. Mass spectra were recorded on BRUKER utilizing ESI as a particle source.

#### 2.4.1. Synthesis of 2-(1(4-chlorobenzoyl),5-methoxy-2-methyl-1*H*-indol-3-yl)-*N*-phenylacetamide **3a**

A total of 0.0022 mol aniline was added to a mixture of 0.002 mol indomethacin, with DMAP and DCM as a solvent. The reaction mixture temperature was maintained at 0 °C while stirring for 30 min. A cooled solution of dicyclohexylcarbodiimide was added to the reaction mixture and stirring continued for 24 h at room temperature. The progress of the reaction was monitored using TLC with 30% ethyl acetate:hexane as the solvent system. The reaction was carried out using DCM followed by a repeated wash with brine and sodium bicarbonate.

#### 2.4.2. Synthesis of 2-(1-(4-chlorobenzoyl),5-methoxy-2-methyl-1*H*-indol-3-yl)-*N*-(2-chlorophenyl)acetamide **3b**

The reaction process performed was the same as for compound **3a**, wherein aniline was replaced with o-chloroaniline (0.002 mol), and the reaction carried out at room temperature gave a yellow colour product of **3b**. 

#### 2.4.3. Synthesis of N-(3-chloro-4-fluorophenyl)-2-(1-(4-chlorobenzoyl)-5-methoxy-2-methyl-1*H*-indol-3-yl)acetamide **3c**

The reaction process performed was the same as for compound **3a**, wherein aniline was replaced with 3-chloro-4-fluoroaniline (0.002 mol), and the reaction carried out at room temperature produced a pale yellow colour product of **3c**.

#### 2.4.4. Synthesis of 2-(1-(4-chlorobenzoyl)-5-methoxy-2-methyl-1*H*-indol-3-yl)-*N*-(4-fluorophenyl)acetamide **3d**

The reaction process performed was the same as for compound **3a**, wherein aniline was replaced with p-fluoroaniline (0.002 mol), and the reaction carried out at room temperature afforded a dark yellow colour product of **3d**.

#### 2.4.5. Synthesis of 2-(1-(4-chlorobenzoyl)-5-methoxy-2-methyl-1*H*-indol-3-yl)-*N*-(4-nitrophenyl)acetamide **3e**

The reaction process performed was the same as for compound **3a**, wherein aniline was replaced with p-nitroaniline (0.002 mol), and the reaction carried out at room temperature gave a florescent yellow colour product of **3e**.

#### 2.4.6. Synthesis of 2-(1-(4-chlorobenzoyl)-5-methoxy-2-methyl-1*H*-indol-3-yl)-*N*-(4-chlorophenyl)acetamide **3f**

The reaction process performed was the same as for compound **3a**, wherein aniline was replaced with 4-chloroaniline(0.002 mol), and the reaction carried out at room temperature produced a whitish yellow colour product of **3f**.

#### 2.4.7. Synthesis of 2-(1-(4-chlorobenzoyl)-5-methoxy-2-methyl-1*H*-indol-3-yl)-*N*-(2,6-dichlorophenyl)acetamide **3g**

The reaction process performed was the same as for compound **3a**, wherein aniline was replaced with 2,6-dichloroaniline(0.002 mol), and the reaction carried out at room temperature produced a white colour product of **3g**.

#### 2.4.8. Synthesis of N-benzyl-2-(1-(4-chlorobenzoyl)-5-methoxy-2-methyl-1*H*-indol-3-yl)acetamide **3h**

The reaction process performed was the same as for compound **3a**, wherein aniline was replaced with benzylamine (0.002 mol), and the reaction carried out at room temperature produced a white colour product of **3h**.

#### 2.4.9. Synthesis of 2-(1-(4-chlorobenzoyl)-5-methoxy-2-methyl-1*H*-indol-3-yl)-*N*-m-tolylacetamide **3i**

The reaction process performed was the same as for compound **3a**, wherein aniline was replaced with p-toluidine (0.002 mol), and the reaction carried out at room temperature produced a pale yellow colour product of **3i**.

### 2.5. Pharmacological Screening

#### 2.5.1. In Vitro COX-1 and COX-2 Enzymatic Assay

In vitro, the inhibitory activity of the synthesized molecules, COX-I and COX-2, were examined using commercially available calorimetric enzyme assay kits (Cayman Chemicals, Ann Arbor, MI, USA) [14]. The assay procedure was followed as per the manufacturer’s protocol with slight modifications. The screening kits consolidate ovine COX-1 as well as human recombinant COX-2 compounds to screen isozyme-specific inhibitors. The test investigates the peroxidase activity by projecting the presence of oxidized TMPD (N, N, N′, N′- tetramethyl-p-phenylenediamine) at 590 nm. The COX-2 selectivity was measured as a ratio of IC50 for COX-1 divided by COX-2 [15].

#### 2.5.2. In Vivo Carrageenan-Induced Rat Paw Oedema Study

The in vivo study protocol reported in this study is supported by the Institutional Animal Ethics Committee (IAEC) of the Institute of Pharmacy, Nirma University, Ahmedabad (vide protocol number IP/PCOL/MPH/17/006). All the procedures performed followed the Committee for Control and Supervision of Experiments on Animals (CPCSEA), the Ministry of Social Justice and Empowerment, the Government of India. The in vivo study was performed using Wistar rats (250–300 gm body weight) obtained from the Torrent Research Center, Gandhinagar.

Animals were divided into five groups containing six rats per group and housed under controlled conditions. The temperature and relative humidity of the animal house were kept at 23 ± 2 °C and 55 ± 5%, respectively. Animals were kept under a photo schedule of 12 h light and 12 h dark cycle and given access to food and purified water ad libitum. Before starting the experiment, all animals were acclimatized for one week and divided randomly into five groups, namely the healthy control group, the disease control group, animals treated with indomethacin (2.57 mg/kg), animals treated with test compounds **3c** (2.57 mg/kg) and **3e** (2.57 mg/kg). A freshly prepared carrageenan solution (1%*w*/*v*, 0.1 mL) was injected into the plantar side of each rat’s right hind paw [16]. All test compounds, including indomethacin, were suspended in 0.5% CMC and were administered orally 1 h before the carrageenan injection. The paw volume was measured using a plethysmometer using the mercury displacement method after 5 h of carrageenan injection.

#### 2.5.3. The Effect of Indomethacin Derivatives on the Gastric Mucosa

GI side effects were assessed six hours after an oral administration in each group. Animals were euthanized, and stomachs were expelled, opened along the more prominent bend and washed with saline to remove gastric contents. Each stomach was analysed by another researcher, blinded to the treatment groups. The stomach was visually examined for the presence of any lesions, hyperaemia (red tinge) or haemorrhagic spots for evaluation of the presence of ulceration.

## 3. Results

### 3.1. Molecular Docking Studies

**Figure 1:**
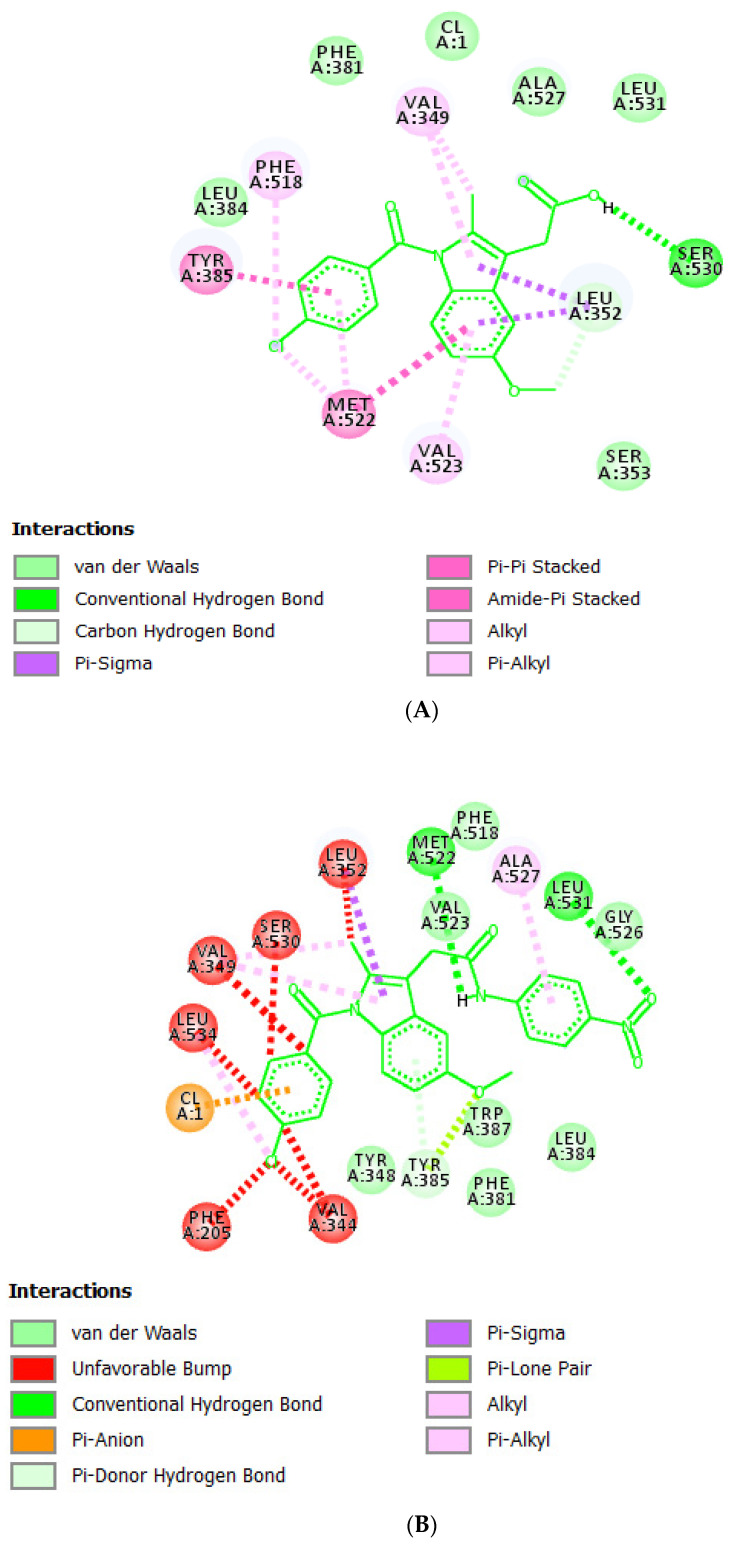
Molecular docking study of heterocyclic compounds. (**A**) Docking of indomethacin with COX-2 enzyme. (**B**) Docking of **3i** with COX-2 enzyme.

Docking was performed using SYBYL X1.2 and GOLD 5.2 suite to analyse the interaction of synthesized derivatives with the COX-2 enzyme. All the derivatives displayed higher or comparable docking scores to standard indomethacin, as depicted in Table 1. The binding affinity of the ligand with the amino acids of COX protein is shown in Figure 1.

### 3.2. Molecular Dynamic Simulation

**Figure 2:**
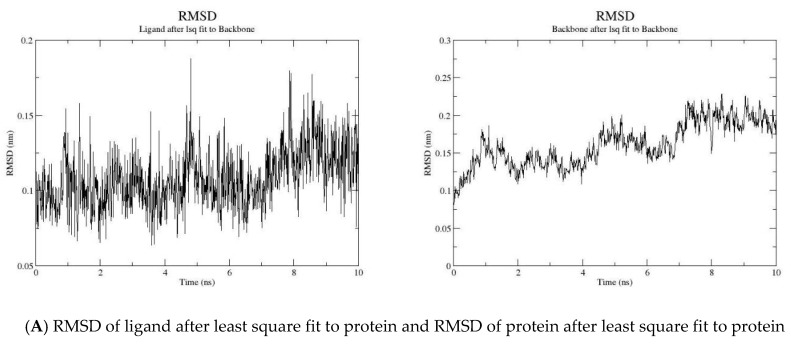

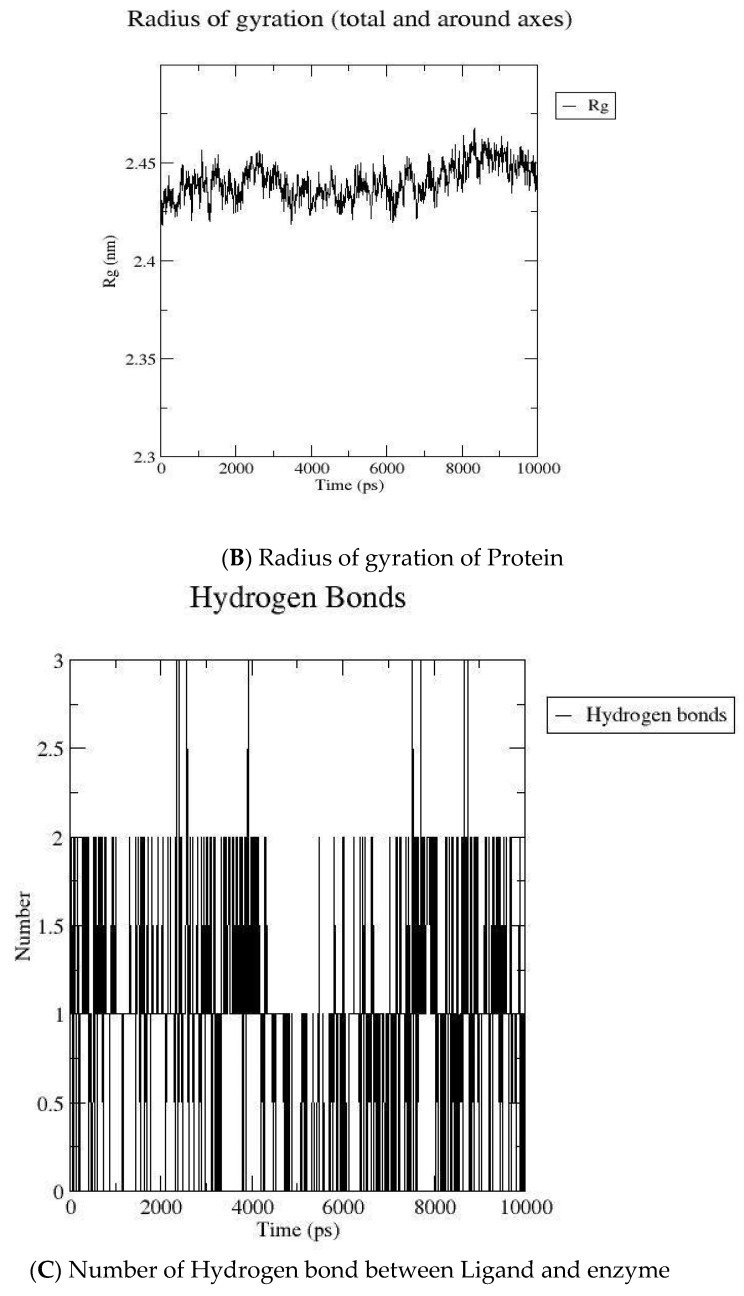

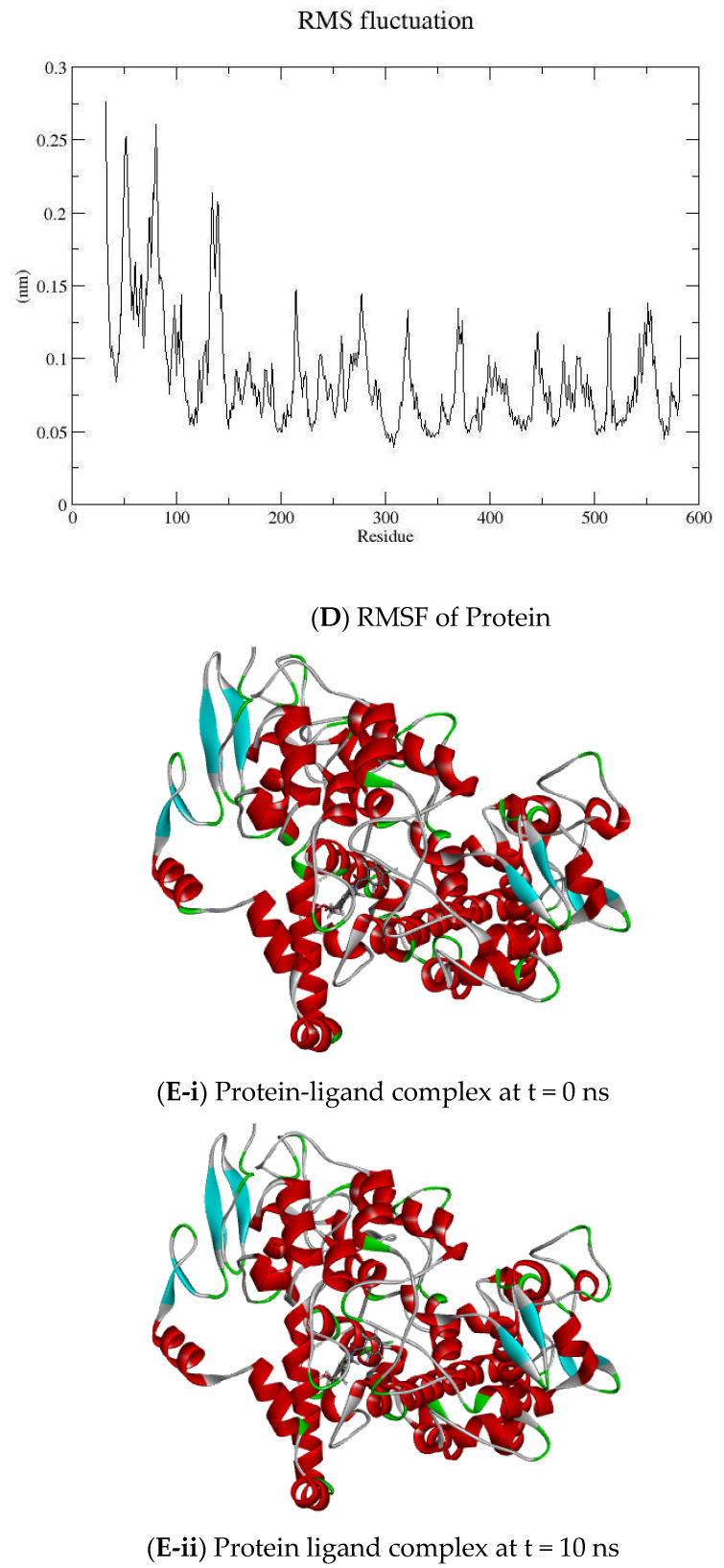
MD simulation (**A**) RMSDs of ligand after least square fit to protein (left) and RMSDs of protein after least square fit to protein (right). (**B**) Radius of gyrations of protein. (**C**) Number of hydrogen bonds formed between ligand and enzyme. (**D**) Results of RMSF analysis of the top-scoring ligand–protein complex. (**E**) The structures of 3NT1–**3e** complex recorded at start (**E-i** at t = 0 ns) and end (**E-ii** at t = 10 ns).

After molecular docking calculations, the top-scoring ligand–protein complex, 3NT1–**3e**, was subjected to 10 ns MD simulations. The radius of gyration and the root mean square deviation (RMSD) are measures of complex stability. The RMSD and radius of gyration were measured in the study to validate the docking positions and structural stability. Throughout the MD simulation, the number of hydrogen bonds was also counted. Figure 2A shows the RMSDs of ligand and the RMSDs of protein backbones after a least-square fit to protein backbones. The results of the 3NT1–**3e** complex demonstrate that the ligand quickly found its equilibrium position in the binding pocket and stayed there throughout the simulation. During the simulation, it was also discovered that the RMSD of protein did not alter significantly.

Another valuable measure for studying protein conformational stability and integrity in ligand–protein interactions is the radius of gyration (Figure 2B). The Rg of the protein was monitored in addition to the number of hydrogen bonds formed between the ligand and the protein, as shown in Figure 2C. According to the findings, there were no substantial changes in the Rg of protein. As a result, it was determined that the protein remained stable throughout the MD simulations. The 3NT1–**3e** complex’s average Rg values and standard deviations were 2.44 nm (0.0091). According to the findings, the average number of hydrogen bonds produced between ligand and enzyme was found to be 1 for the 3NT1–**3e** complex. As a result, it was shown that hydrogen bonding van der Waals interactions were the most common in the protein–ligand combination. Figure 2D shows the root mean square fluctuations (RMSF) of the residues for all top-scoring ligand–protein complexes. The RMSF of the residues positioned in the binding pocket and involved in ligand–protein interactions were found to be lower in the 3NT1–**3e** complex than in the comparable uninhibited enzyme, according to RMSF studies. Figure 2E show the structures of the top-scoring ligand–protein complex retrieved from the simulation trajectories. The examined ligand was bound to the enzyme’s active site throughout the simulation and remained confined in the binding pocket.

### 3.3. LD_50_ Predictions Using GUSAR

The acute toxicity of the designed molecules was predicted using the free online GUSAR software. The prediction of the LD50 value for different routes of administration such as intravenous (IV), oral, and intraperitoneal (IP) are shown in Table 2. The predicted values revealed that most of the compounds were found to be under the BCS class 4 category. All compounds were out of the applicability domain except **3c**, **3d** and **3i** using the subcutaneous route. Additionally, compound **3c** was nontoxic while considering the subcutaneous route. All the compounds were found to be in the applicability domain using intravenous (IV), oral, and intraperitoneal (IP) routes [13].

### 3.4. Physical Characterization of Indomethacin Derivatives

**Scheme 1:**
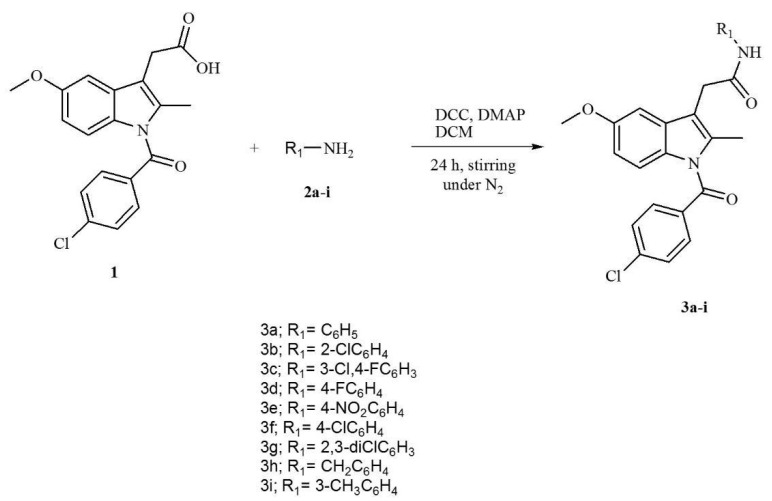
Synthetic scheme for the target compounds.

Indomethacin derivatives were synthesized using DCC/DMAP coupling utilizing substituted aromatic amines (Scheme 1). The structural characterization of indomethacin derivatives was performed utilizing IR, mass, and NMR (both ^1^H NMR and ^13^C-NMR) spectroscopy [17,18].

#### 3.4.1. Structural Characterization of 2-(1(4-chlorobenzoyl),5-methoxy-2-methyl-1*H*-indol-3-yl)-*N*-phenylacetamide **3a**

32% yield, m.p. 160–165°C, ^1^H NMR (400 MHz, CDCl_3_) δ PPM: 7.7 (d, *J* = 8.48 Hz, 2H) 7.51 (d, *J* = 8.48 Hz, 2H) 7.37 (d, *J* = 7.8 Hz, 2H) 7.28 (t, *J* = 7.4 Hz, 2H) 7.09 (t, *J* = 7.36 Hz, 1H) 6.95 (d, 1H) 6.88 (s, 1H) 6.86 (s, 1H) 6.72 (d, 1H) 6.70 (d, 1H) 3.81 (s, 2H) 2.45 (s, 3H) 2.30 (s, 3H) ^13^C NMR (400 MHz, CDCl_3_) δ PPM: 168.36, 168.14, 156.40, 139.71, 137.33, 136.71 (2C), 133.47, 131.26 (2C), 130.94, 130.11, 129.29, 129.00, 124.68, 120.14, 115.26, 112.56, 112.34, 100.63, 55.78, 33.9, 25.6. ESI-MS: *m*/*z* = 434 [M + 2]^+^.

#### 3.4.2. Structural Characterization of 2-(1-(4-chlorobenzoyl),5-methoxy-2-methyl-1*H*-indol-3-yl)-*N*-(2-chlorophenyl)acetamide **3b**

45.4% yield, m.p. 170–172 °C, ^1^H NMR (400 MHz, CDCl_3_) δ PPM: 7.89 (d, 2H) 7.87 (d, 2H) 7.65 (d, *J* = 7.7 Hz, 2H) 7.57 (t, *J* = 7.8 Hz, 1H) 7.45 (d, 1H) 6.95 (s, 1H) 6.81 (s, 1H) 6.77 (d, 1H) 6.72 (d, 1H) 3.63 (s, 2H) 2.23 (s, 3H) 2.14 (s, 3H) ^13^C NMR (400 MHz, CDCl_3_) δ PPM: 168.11, 167.8, 151.34, 148.38, 137.67, 137.18 (2C), 133.13, 131.41 (2C), 130.43, 130.23, 129.54, 128.65, 124.88, 122.00, 115.01, 112.63, 109.71, 105.27, 50.68, 28.68, 19.62. ESI-MS: *m*/*z* = 468 [M + 1]^+^.

#### 3.4.3. Structural Characterization of N-(3-chloro-4-fluorophenyl)-2-(1-(4-chlorobenzoyl)-5-methoxy-2-methyl-1*H*-indol-3-yl)acetamide **3c**

68.3% yield, m.p. 140–145 °C, ^1^H NMR (400 MHz, CDCl_3_) δ PPM: 7.78 (d, *J* = 7.8 Hz, 2H) 7.63 (d, *J* = 7.8 Hz, 2H) 7.60 (d, *J* = 8.4 Hz, 2H) 7.51 (d, *J* = 7.7 Hz, 1H) 7.41 (d, *J* = 8.41 Hz, 1H) 7.25 (s, 1H) 6.88 (d, *J* = 2.4 Hz, 1H) 6.79 (d, *J* = 8.8 Hz, 1H) 6.61 (d, *J* = 6.8 Hz, 1H) 3.76 (s, 2H) 3.58 (s, 3H) 2.31 (s, 3H) ^13^C NMR (400 MHz, CDCl_3_) δ PPM: 174.24, 171.37, 168.32, 156.04, 139.28, 135.99 (2C), 133.89, 131.19 (2C), 130.79, 130.65, 129.14, 128.95, 125.63, 123.48, 114.97, 112.51, 111.61, 101.28, 55.73, 33.83, 24.91. ESI-MS: *m*/*z* = 486 [M + 1]^+^.

#### 3.4.4. Structural Characterization of 2-(1-(4-chlorobenzoyl)-5-methoxy-2-methyl-1*H*-indol-3-yl)-*N*-(4-fluorophenyl)acetamide **3d**

65.2% yield, m.p. 155–160 °C, ^1^H NMR (400 MHz, CDCl_3_) δ PPM: 7.52 (d, *J* = 7.3 Hz, 2H) 7.35 (d, *J* = 7.7 Hz, 2H) 7.22 (d, *J* = 7.7 Hz, 2H) 7.18 (t, *J* = 8 Hz, 1H) 7.1 (d, 1H) 6.86 (s, 1H) 6.47 (s, 1H) 6.32 (d, 1H) 6.28 (d, 1H) 3.54 (s, 2H) 2.36 (s, 3H) 2.25 (s, 3H) ^13^C NMR (400 MHz, CDCl_3_) δ PPM: 169.67, 168.8, 154.76, 145.26, 139.46, 136.62 (2C), 134.18, 130.68 (2C), 130.2, 129.89, 128.14, 128.97, 125.74, 122.37, 116.08, 114.32, 107.62, 103.48, 55.23, 25.69, 20.47. ESI-MS: *m*/*z* = 452 [M + 2]^+^.

#### 3.4.5. Structural Characterization of 2-(1-(4-chlorobenzoyl)-5-methoxy-2-methyl-1*H*-indol-3-yl)-*N*-(4-nitrophenyl)acetamide **3e**

50.2% yield, m.p. 165–170 °C, ^1^H NMR (400 MHz, CDCl_3_) δ PPM: 8.04 (d, 2H) 7.57 (d, 2H) 7.40 (s, 1H) 6.53–6.88 (m, 6H) 4.47 (s, 1H) 3.58 (s, 3H) 2.31 (s, 2H) 1.86 (s, 3H) ^13^C NMR (400 MHz, CDCl_3_) δ PPM: 2.31 (s, 2H). ESI-MS: *m*/*z* = 479 [M + 1].

#### 3.4.6. Structural Characterization of 2-(1-(4-chlorobenzoyl)-5-methoxy-2-methyl-1*H*-indol-3-yl)-*N*-(4-chlorophenyl)acetamide **3f**

68.6% yield, m.p. 160–162 °C, ^1^H NMR (400 MHz, CDCl_3_) δ PPM: 8.18 (d, 7.7 Hz 1H) 7.83 (d, J= 7.7 Hz 1H) 7.47 (m, 6H) 6.68 (s, 1H) 6.49 (d, *J* = 7.8 Hz, 2H) 6.02 (s, 1H) 3.56(s, 3H) 2.53 (s, 2H) 2.30 (s, 3H) ^13^C NMR (400 MHz, CDCl_3_) δ PPM: 168.36, 167.93, 151.66, 153.76, 149.12, 147.83, 143.12, 142.56, 141.17, 140.67, 138.51, 134.35, 132.23, 129.85, 126.55, 124.34, 121.04, 120.42, 119.53, 115.38, 110.67, 100.78, 50.56, 23.47, 14.78. ESI-MS: *m*/*z* = 468 [M + 1]^+^.

#### 3.4.7. Structural Characterization of 2-(1-(4-chlorobenzoyl)-5-methoxy-2-methyl-1*H*-indol-3-yl)-*N*-(2,6-dichlorophenyl)acetamide **3g**

58.11% yield, m.p. 150–155 °C, ^1^H NMR (400 MHz, CDCl_3_) δ PPM: 7.66 (d, *J* = 8.44 Hz, 2H) 7.48 (d, *J* = 8.36 Hz, 2H) 7.39 (m, 4H) 7.29 (s, 1H) 6.93 (d, 1H) 6.72 (d, 1H) 3.9 (s, 3H) 3.4 (s, 2H) 2.3 (s, 3H) ^13^C NMR (400 MHz, CDCl_3_) δ PPM: 168.37, 168.29, 156.80, 156.39, 139.75, 136.76(2C), 133.41, 131.91(2C), 130.94(2C), 130.09, 129.30(2C), 121.67, 121.49, 117.23, 115.25, 112.43, 112.13, 100.71, 55.79, 33.29, 24.95. ESI-MS: *m*/*z* = 504 [M + 2]^+^.

### 3.5. Biological Screening

#### 3.5.1. In Vitro Biological Screening of Indomethacin Analogues

The in vitro screening revealed that compounds **3c** and **3e** were more selective towards COX-2 enzyme with an IC_50_ value of 1.39 µM and 0.344 µM, respectively, compared with indomethacin. Compound **3e** showed comparable COX-2 inhibition potential to indomethacin. The selectivity ratio (ratio of IC50 for COX-1 to COX-2) of **3c** was less than indomethacin, while compound **3e** had similar selectivity but more potency than indomethacin, as depicted in Table 3.

#### 3.5.2. In Vivo Pharmacological Screening

The compounds with a better in vitro evaluation profile were subjected to in vivo pharmacological screening. The paw volume of the disease control group increased to about 58% after five hours compared with the control group animals.

**Figure 3:**
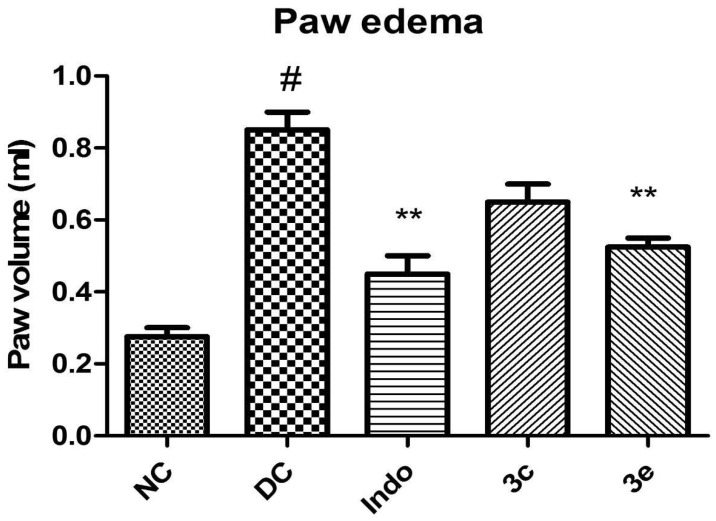
Effect of heterocyclic derivatives on animal model of carrageenan-induced paw oedema after 5 h of induction. # Significantly different from normal control group (*p* < 0.001), ** significantly different from disease control group (*p* < 0.05). Each group consisted of 6 animals. Values are expressed as mean ± SEM. NC, normal control; DC, disease control; Indo, disease treated with indomethacin (2.57 mg/kg); **3c**, disease treated with **3c** (2.57 mg/kg); **3e**, disease treated with **3e** (2.57 mg/kg).

The in vivo study results revealed the significant inhibition of oedema formation in the rats treated with synthesized compounds and standard indomethacin, as shown in Figure 3. The study revealed a similar anti-inflammatory activity of compound **3e**, an indomethacin derivative substituted with a p-nitrophenyl ring. 

#### 3.5.3. Determination of Ulcerogenic Effect

**Figure 4:**
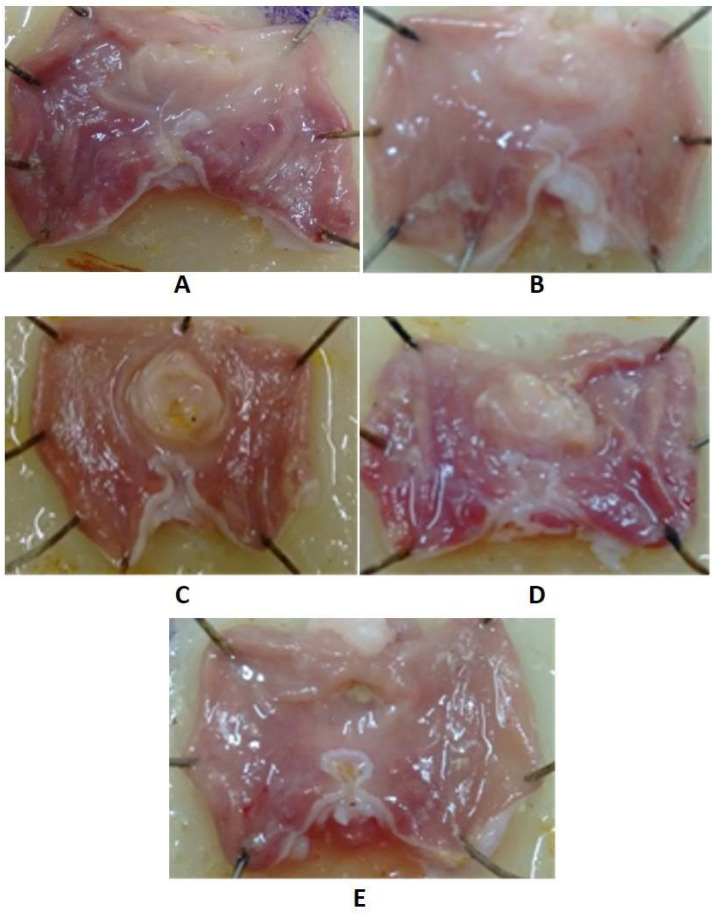
Effect of indomethacin derivatives on peptic ulcer. (**A**) Normal control group, (**B**) disease control group, (**C**) disease treated with indomethacin (2.57 mg/kg), (**D**) disease treated with **3c** (2.57 mg/kg), (**E**) disease treated with **3e** (2.57 mg/kg).

For the evaluation of the ulcerogenic effect, the stomach tissues were visually examined for the presence of lesions, red colouration, haemorrhagic spots and ulcers. No lesions or ulcerations were found in the heterocyclic derivative-treated group six hours after administration, while animals treated with indomethacin showed small lesions and red colouration, as shown in Figure 4.

## 4. Discussion

Cyclooxygenase is an essential enzyme required to synthesize prostaglandins, thromboxane and leukotriene from arachidonic acid. The COX-1 enzyme is constitutive and responsible for synthesizing prostaglandins that control renal function, platelet aggregation, and gastro protection. The COX-2 enzyme is induced during injury and leads to the synthesis of some prostanoids, which mediate actions such as pain, inflammation, fever and the inhibition of platelet aggregation. Many research groups mainly focus on the synthesis of selective COX-2 inhibitors. The heterocyclic inhibitors of the COX-2 enzyme were synthesized to decrease gastrointestinal adverse effects [19]. However, the inhibition of COX-1 and COX-2 enzymes leads to adverse events, while the inhibition of COX-2 enzymes is selectively attributed to increased risk of cardiovascular side effects. Hence, uncertainty still surrounds using selective COX-2 inhibitors because of their cardiovascular and thrombotic adverse events [20]. The main goal of the research was to synthesize potential derivatives of the classical NSAIDs while considering the side effects associated with the inhibition of COX-1 and COX-2 enzymes. Researchers have been preparing different esters or amide derivatives of anti-inflammatory agents. As a result of the research, indomethacin analogues have been developed. Substituted aromatic amines were used in the development of indomethacin amide derivatives. The heterocyclic compounds were developed to have a potent pharmacological effect with minimal side effects.

All the synthesized novel compounds displayed hydrogen bonding interaction with the amino acids of the COX-2 protein during the molecular docking study. All compounds showed comparable or higher docking scores than the standard indomethacin. PASS online software was used to predict the in silico toxicity of the designed molecules. Most of the compounds were found in the applicability domain of the QSAR models. The toxicity predictions considered using the IV, oral, subcutaneous and intraperitoneal routes, which revealed that all the compounds fall in class 4. According to the study, the compounds are slightly toxic.

The amide derivatives of indomethacin were synthesized using well-established methods, as shown in Scheme 1. Various amide derivatives of indomethacin were synthesized using amide coupling, utilizing different aromatic amines. Treatment with the appropriate aromatic amines in the presence of dicyclohexylcarbodiimide (DCC) and 4-dimethylaminopyridine (DMAP) yielded indomethacin derivatives. The yield of halogen-substituted phenyl derivatives was found to be higher as compared with the substituted phenyl ring. The yield of 2-chloro substituted amine was found to be lower as compared with the substituted and 4-chloro substituted derivatives.

In vitro, COX-1 and COX-2 colorimetric assays were performed on the synthesized compounds. Except for **3f**, **3h**, and **3i**, all compounds had similar inhibitory efficacy against the COX-2 enzyme. The selectivity for the COX-2 enzyme was reduced when the phenyl ring was substituted with chlorine, as in the case of **3f**. In addition, phenyl replaced with an electron-donating group such as methyl had low inhibitory action against COX-2, as in the case of **3h** and **3i**. Compared with other derivatives, compound **3e** (phenyl ring substituted with the nitro group) demonstrated the highest selectivity ratio.

In addition, as compared with the standard, compound **3e** had particularly significant activity against the COX-2 enzyme. There are many pharmaceutical industry efforts to discover selective COX-2 inhibitors with fewer side effects. Efforts have been made to develop anti-inflammatory agents devoid of adverse cardiovascular events [5]. The study represents the design, synthesis and in vitro and in vivo biological screening of heterocyclic derivatives. The compounds that displayed promising results during in vitro testing were subjected to in vivo screening. The in vivo anti-inflammatory response of synthesized molecules was measured using the carrageenan-induced rat paw oedema method. Both the selected compounds produced comparable anti-inflammatory activity with the standard in the working model of inflammation. 

In vivo pharmacological evaluation of synthesized compounds was performed using the carrageenan-induced rat paw oedema method. During the in vivo study, both compounds (**3c** and **3e**) demonstrated comparable anti-inflammatory activity with the standard. The mechanism behind the anti-inflammatory activity is expected to interact with the mediators of inflammation, e.g., prostaglandins and cytokines. Additionally, carrageenan activates macrophages and polymorphonuclear cells. Hence, COX-2 enzyme inhibition is essential for the anti-inflammatory activity of compounds under screening [21]. NSAIDs inhibit the synthesis of prostaglandins required for gastric acid secretion and gastroprotection. There are reports which support the use of NSAIDs involved in the origin of gastric ulcers [22]. The ulcer index was calculated in the present study, and the stomach was analysed for ulcers. The results indicate indomethacin with small lesions and ulcers, while the synthesized derivatives were devoid of any ulcerogenic effects in the treated rats. Thus, **3e** has anti-inflammatory activity equal to indomethacin but is devoid of GI side effects such as ulceration. From the present study, it is revealed that selective COX-2 inhibitors do not affect the gastrointestinal tract mucosa significantly, and derivatives have been developed as gastrointestinal-sparing anti-inflammatory drugs.

## 5. Conclusions

COX-2 inhibitors are being used widely and several reports have documented trying to resolve the issues related to NSAIDs’ associated toxicity. Indomethacin derivatives were designed and synthesized using amide coupling utilizing various aromatic amines. Docking studies revealed that all the compounds showed a high docking score and binding affinity with the active site amino acids. The MD simulation results demonstrated that the ligand in the 3NT1–**3e** ligand–protein complex was efficiently bound to the protein’s active site and remained bound in the binding pocket throughout the simulation. According to the acute toxicity predictions, all the compounds were safe. The synthesis includes one step of acid amide coupling. In vitro screening of the derivatives demonstrated compounds **3c** and **3e** to possess promising COX-2 inhibitory activity. Compound **3e** showed an excellent selectivity ratio as compared with the standard. In a nutshell, indomethacin analogues substituted with a p-nitrophenyl ring were found to have potential anti-inflammatory activity and can be further evaluated to control different inflammatory diseases.

## Figures and Tables

**Table 1:** Molecular docking study of heterocyclic compounds.

Compound Code	GOLD Score	Interaction
Indomethacin	45.07	Val523, Ser530, Tyr385
**3a**	50.08	Ser530, Tyr385
**3b**	51.09	Tyr385, Trp387
**3c**	49.66	Val 523, Ser 530
**3d**	46.55	Ser530, Tyr385
**3e**	48.11	Trp 387, Ser 530
**3f**	48.03	Val 523, Ser 530
**3g**	47.34	Leu 384, Val 523
**3h**	45.06	Ser530, Tyr385
**3i**	57.88	Val 523, Ser 530, Tyr 385 Trp 387, Leu 384

**Table 2:**
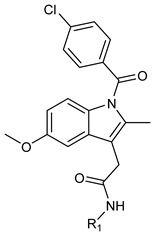
Acute toxicity prediction using GUSAR software.

Compound Code	R_1_	LD_50_ (mg/kg)			
		IP	IV	Oral	SC
Indomethacin	-	62.3 ^b^ *	60.9 ^a #^	80.8 ^b^ *	50.8 ^b^ *
**3a**	C_6_H_5_	347.7 ^a #^	82.85 ^a #^	452.7 ^a #^	449.8 ^b #^
**3b**	2-ClC_6_H_4_	312.4 ^a #^	36.64 ^a^ *	391.6 ^a #^	318.7 ^b #^
**3c**	3-Cl,4-FC_6_H_3_	271 ^a #^	60.33 ^a #^	458.6 ^a #^	2870 ^a $^
**3d**	4-FC_6_H_4_	309.2 ^a #^	43.26 ^a #^	344.6 ^a #^	2245 ^a &^
**3e**	4-NO_2_C_6_H_4_	384.7 ^a #^	44.16 ^a #^	417.5 ^a #^	227.3 ^b #^
**3f**	4-ClC_6_H_4_	322.6 ^a #^	45..7 ^a #^	546.2 ^a #^	343.6 ^b #^
**3g**	2,3-dichloroC_6_H_3_	260.3 ^a #^	44.23 ^a #^	403.7 ^a #^	347.8 ^b #^
**3h**	CH_2_C_6_H_5_	414.5 ^a #^	71.13 ^a #^	337.8 ^a #^	374.7 ^b #^
**3i**	3-CH_3_C_6_H_4_	334.4 ^a #^	45.68 ^a #^	480.6 ^a #^	1494 ^a &^

IP, intraperitoneal route; IV, intravenous route; SC, subcutaneous route; oral, ^&^ Compound falls in class 5, ^#^ compound falls in class 4, * compound falls in class 3, ^$^ compound is nontoxic. Applicability Domain: ^a^ compound falls in applicability domain models; ^b^ compound is out of applicability domain of models.

**Table 3:** IC_50_ value of indomethacin derivatives.

Compound Code	IUPAC Name	IC_50_ COX-2 (µM)	IC_50_ COX-1 (µM)	Selectivity
Indomethacin	2-(1-(4-chlorobenzoyl)-5-methoxy-2-methyl-1-*H*-indol-3-yl)acetic acid	1.06 ± 0.31	3.50 ± 0.05	3.29
**3a**	2-(1(4-chlorobenzoyl),5-methoxy-2-methyl-1*H*-indol-3-yl)-*N*-phenylacetamide	2.97 ± 0.11	6.04 ± 0.14	2.04
**3b**	2-(1-(4-chlorobenzoyl),5-methoxy-2-methyl-1*H*-indol-3-yl)-*N*-(2-chlorophenyl)acetamide	3.28 ± 0.16	4.61 ± 0.31	1.41
**3c**	N-(3-chloro-4-fluorophenyl)-2-(1-(4-chlorobenzoyl)-5-methoxy-2-methyl-1*H*-indol-3-yl)acetamide	1.39 ± 0.32	1.06 ± 0.10	0.76
**3d**	2-(1-(4-chlorobenzoyl)-5-methoxy-2-methyl-1*H*-indol-3-yl)-*N*-(4-fluorophenyl)acetamide	2.45 ± 0.03	5.0 ± 0.47	2.04
**3e**	2-(1-(4-chlorobenzoyl)-5-methoxy-2-methyl-1*H*-indol-3-yl)-*N*-(4-nitrophenyl)acetamide	0.34 ± 0.31	1.15 ± 0.30	3.32
**3f**	2-(1-(4-chlorobenzoyl)-5-methoxy-2-methyl-1*H*-indol-3-yl)-*N*-(4-chlorophenyl)acetamide	4.71 ± 0.4	3.55 ± 0.2	0.75
**3g**	2-(1-(4-chlorobenzoyl)-5-methoxy-2-methyl-1*H*-indol-3-yl)-*N*-(2,6-dichlorophenyl)acetamide	2.91 ± 0.38	4.14 ± 0.25	1.42
**3h**	N-benzyl-2-(1-(4-chlorobenzoyl)-5-methoxy-2-methyl-1*H*-indol-3-yl)acetamide	4.73 ± 0.15	4.99 ± 0.11	1.06
**3i**	2-(1-(4-chlorobenzoyl)-5-methoxy-2-methyl-1*H*-indol-3-yl)-*N*-m-tolylacetamide	4.22 ± 0.23	4.71 ± 0.22	1.12

## Data Availability

Not applicable.
